# Amino Terminal Domains of the NMDA Receptor Are Organized as Local Heterodimers

**DOI:** 10.1371/journal.pone.0019180

**Published:** 2011-04-22

**Authors:** Chia-Hsueh Lee, Eric Gouaux

**Affiliations:** 1 Vollum Institute, Oregon Health and Science University, Portland, Oregon, United States of America; 2 Howard Hughes Medical Institute, Oregon Health and Science University, Portland, Oregon, United States of America; German Cancer Research Center, Germany

## Abstract

The *N*-methyl-D-aspartate (NMDA) receptor, an obligate heterotetrameric assembly organized as a dimer of dimers, is typically composed of two glycine-binding GluN1 subunits and two glutamate-binding GluN2 subunits. Despite the crucial role that the NMDA receptor plays in the nervous system, the specific arrangement of subunits within the dimer-of-dimer assemblage is not conclusively known. Here we studied the organization of the amino terminal domain (ATD) of the rat GluN1/GluN2A and GluN1/GluN2B NMDA receptors by cysteine-directed, disulfide bond-mediated cross-linking. We found that GluN1 ATDs and GluN2 ATDs spontaneously formed disulfide bond-mediated dimers after introducing cysteines into the L1 interface of GluN2A or GluN2B ATD. The formation of dimer could be prevented by knocking out endogenous cysteines located near the L1 interface of GluN1. These results indicate that GluN1 and GluN2 ATDs form local heterodimers through the interactions in the L1-L1 interface and further demonstrate a dimer-of-heterodimer arrangement in GluN1/GluN2A and GluN1/GluN2B NMDA receptors.

## Introduction

The *N*-methyl-D-aspartate (NMDA) receptor, one subgroup of the ionotropic glutamate receptor family, plays an essential role in neurophysiological functions as well as in neuronal disorders [1], [2]. NMDA receptors are obligate heteromers consisting of two essential GluN1 (NR1) subunits with two GluN2 (NR2) or GluN3 (NR3) subunits [3]. Eight splice-variant isoforms of GluN1, four isoforms of GluN2 (GluN2A–D), and two isoforms of GluN3 (GluN3A–B) have been identified [1], [2]. Each subunit shares a modular domain architecture: an amino terminal domain (ATD) followed by a ligand-binding domain (LBD), a transmembrane domain and a carboxyl terminal domain. Although there is a consensus that the NMDA receptor forms a hetero-tetramer with subunits arranged as a dimer-of -dimers [4], the subunit arrangement within a dimer and the organization of the two dimers are under debate. Two possible arrangements have been proposed. On the one hand, experiments utilizing fluorescence resonance energy transfer (FRET) measurements with fluorophore-tagged subunits and cysteine knockout mutants of GluN1 were interpreted in terms of a dimer of homodimers [5], [6]. On the other hand, the crystal structure of a GluN1/GluN2A LBD heterodimer, FRET measurements with fluorophore-tagged LBDs, and studies on GluN3 subunits are consistent with a dimer-of-heterodimer configuration [7], [8], [9].

To probe the arrangement of NMDA receptor subunits within a dimer, and to resolve the controversy of homodimer versus heterodimer, we studied the association of subunits at the level of the ATD using cysteine-directed chemical cross-linking. The current model for the NMDA receptor ATD has been derived from studies on metabotropic glutamate receptors [10], on non-NMDA ionotropic glutamate receptors, including the GluA2 AMPA receptor and the GluK2 kainate receptor ATD [11], [12], [13], [14], and on the GluN2B ATD [15]. The structures of GluA2 and GluK2 ATDs revealed that the ATD forms a homodimer through the interaction between the L1-L1 domain and L2-L2 domain interfaces [11], [12], [13], [14]. Do NMDA receptor ATDs form architecturally similar local dimers? If the NMDA receptor ATD is indeed a dimer, there are a number of important questions. First, is the dimer a GluN1-GluN1 or GluN2-GluN2 homodimer or is it a GluN1–GluN2 heterodimer? Second, by what interactions does the dimerization occur? For example, do both the L1-L1 and L2-L2 interfaces contribute to the dimerization or does one predominate? Moreover, because the dimerization of ATDs may be an important initial step along the tetrameric assembly pathway of the NMDA receptor [16] and because the ATDs modulate the gating activity of the receptor [17], [18], probing the precise mode of ATD association will help in understanding the principles of NMDA receptor assembly and gating. Here we demonstrate that GluN1 and GluN2 ATDs, in the context of an intact NMDA receptor, form a local heterodimer and that the L1-L1 interface mediates interactions between the ATDs of GluN1 and GluN2 subunits.

## Results

On the basis of amino acid sequence alignments and the structures of the GluA2, GluK2 and the GluN2 ATDs [11], [12], [13], [14], [15], we selected several residues in the putative L1-L1 and L2-L2 ATD dimer interfaces (Fig. S1) for substitution by cysteine because we speculated that these residues may be near each other in an intact NMDA receptor. We asked if any of the introduced cysteine residues would spontaneously form a disulfide bond under ambient conditions. Formation of disulfide bonds would be inferred by the formation of redox-dependent ‘dimer bands’ at an appropriate molecular mass on a sodium dodecyl sulfate polyacrylamide gel. We then employed Western blotting with a GFP antibody to illuminate all NMDA receptor subunits, or with subunit specific antibodies to unambiguously define a particular subunit.

### Cross-linking between NMDA receptor ATDs in the L1-L1 interface

**Figure 1:**
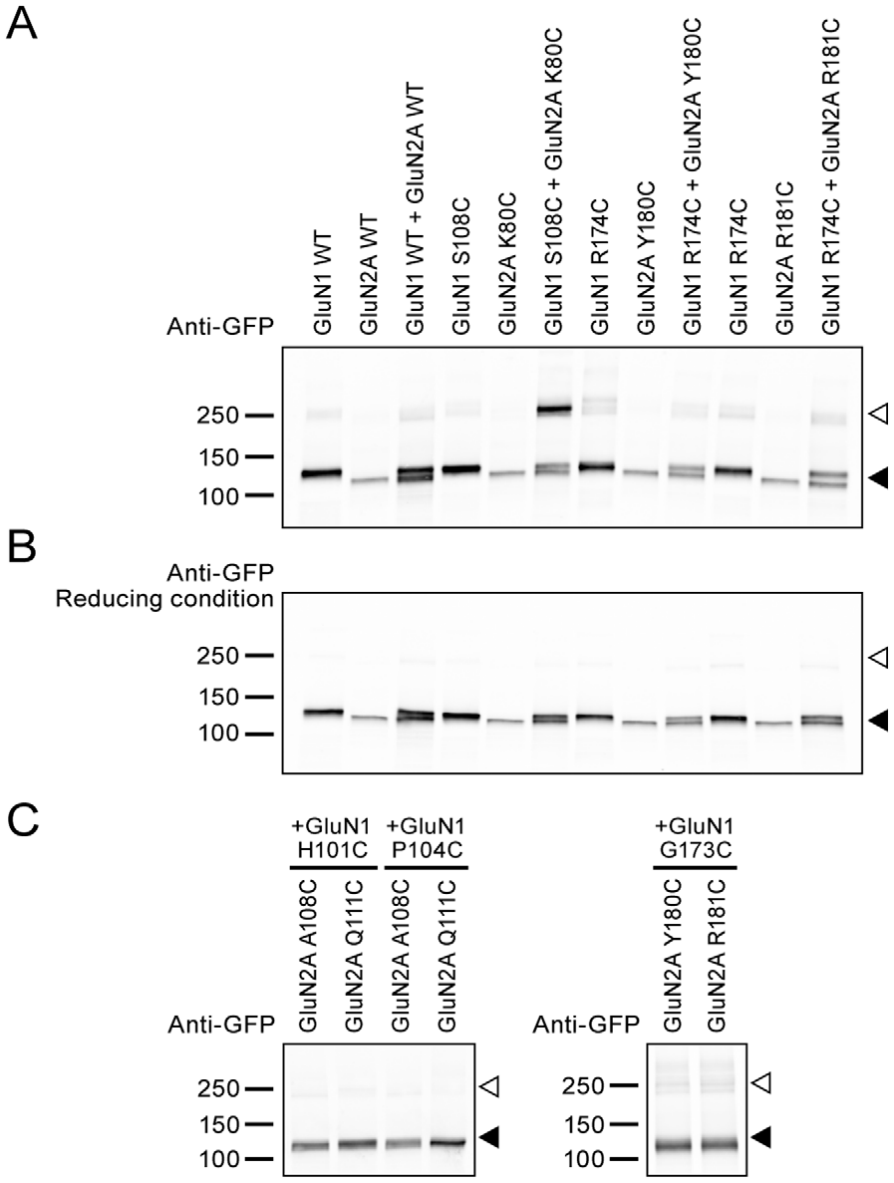
NMDA receptor ATD cysteine mutants in the L1-L1 interface cross-link spontaneously. Western blotting analysis of wild type (WT) or cysteine mutants of NMDA receptor probed by GFP antibody. (**A** and **C**) Non-reducing conditions. (**B**) Reducing conditions. Open arrowhead indicates the cross-linked dimer bands (∼250 kDa). Filled arrowhead indicates positions of GluN1 or GluN2A monomer bands. The predicted molecular weights of GluN1 and GluN2A construct are both ∼125 kDa. However, occasionally we could separate two discrete monomer bands in our gels, probably due to differential glycosylation.

In the GluA2 and GluK2 ATD structures, the L1-L1 interface is defined mostly by helices α2 and α3 (or helices B and C) [11], [12], [13] with several polar and non-polar amino acids participating in the inter-subunit contacts. Among them, a phenylalanine in helix α2, F50 in GluA2 or F58 in GluK2, is conserved through GluA1–A4 to GluK1–K2 and interacts with residues on helix α3 of an adjacent subunit. Moreover, when mutations are introduced into this position, they disrupt the dimerization of the ATD [13]. We therefore mutated the equivalent residue in GluN2A, K80, and its possible interacting partner, to cysteine. When two L1 interface mutants, GluN1 S108C and GluN2A K80C, were coexpressed, a band with higher molecular weight was detected with a mass approximately corresponding to two NMDA receptor subunits (Fig. 1A). Coexpression of wild-type (WT) subunits did not give rise to a similar band. Most importantly, the apparent dimer formed spontaneously without the need of oxidizing reagents or bifunctional cross-linkers. We could abolish the dimer formation by incubation with DTT (Fig. 1A and 1B). The sensitivity of the dimer to reducing agent suggested that dimerization is likely due to formation of a redox-sensitive disulfide bond.

 To determine whether other amino acids that are predicted to reside in a putative L1-L1 interface could also spontaneously give rise to disulfide bond-mediated dimers, we coexpressed a panel of cysteine mutant combinations (Fig. S1): GluN1(H101C)/GluN2A(A108C), GluN1(H101C)/GluN2A(Q111C), GluN1 (P104C)/GluN2A(A108C) or GluN1(P104C)/GluN2A(Q111C). Because none of these cysteine mutant combinations gave rise to a dimer band on the Western blot (Fig. 1C), we believed that the L1-L1 dimer interface was well defined and only specific cysteine residues allowed for spontaneous disulfide bond formation.

We next asked whether we could detect subunit-subunit interactions at the putative L2-L2 dimer interface, drawing upon the recently determined non-NMDA receptor ATD crystal structures as guides. For example, in the GluK2 ATD, L151C on helix F of the L2 interface forms a cross-linked dimer [19]. We thus examined the equivalent residues on the GluN1 and GluN2A ATDs (Fig. S1). Intriguingly, no cross-linked dimer was found when coexpressing GluN1(R174C)/GluN2A(Y180C), GluN1(R174C)/GluN2A(R181C) (Fig. 1B), GluN1(G173C)/GluN2A(Y180C) or GluN1(G173C)/GluN2A(R181C) (Fig. 1C). We therefore concluded that there are significant differences between the L2-L2 interactions in non-NMDA and NMDA receptors.

### GluN1 and GluN2A ATD heterodimer

**Figure 2:**
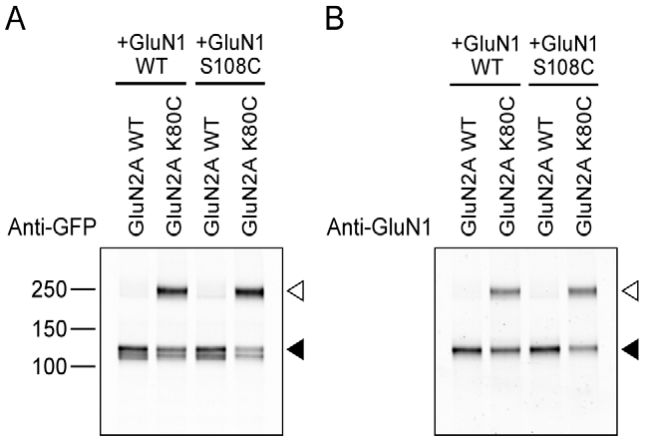
Cross-linking suggests heterodimer formation between GluN1 and GluN2A ATD. Western blotting analysis of GluN2A K80C mutant coexpressed with GluN1 WT or GluN1 S108C mutants. (**A**) The blot probed by GFP antibody. (**B**) The blot probed by GluN1 antibody. Open arrowhead indicates the cross-linked dimer bands. Filled arrowhead indicates positions of GluN1 or GluN2A monomer bands.

We were interested in whether the formation of the putative ATD dimer, as defined by the GluN1 S108C and GluN2A K80C cross-linking, required both engineered cysteine residues. We therefore coexpressed wild-type and cysteine mutants of GluN1 and GluN2A. Surprisingly, we found that dimer formation did not depend on the GluN1 S108C mutant, although the presence of a cysteine residue at S108 modestly enhanced dimer formation. In fact, the coexpression of GluN1 wild-type with GluN2A K80C was sufficient for producing cross-linked dimers (Fig. 2A). This observation suggested two possibilities: either GluN2A K80C cross-linked with an endogenous cysteine on the GluN1 ATD to form a GluN1–GluN2A heterodimer, or GluN2A K80C cross-linked with another GluN2A subunit to form a GluN2A-GluN2A homodimer. If the former hypothesis was correct, then the GluN1 subunit must be present in the dimer band. On the other hand, if GluN1 cannot be detected in the dimer band, then the dimer band was likely the consequence of a GluN2A-GluN2A homodimer. To distinguish between these scenarios, we probed the blot with a GluN1 specific antibody and robustly detected the presence of the GluN1 subunit (Fig. 2B). Furthermore, when the dimer was formed, depletion of the GluN1 and GluN2A monomer populations were almost equal. These data support the conclusion that the GluN1–GluN2A NMDA receptor ATD is a heterodimer and that the GluN2A K80C mutant can form a disulfide bridge with an unidentified endogenous cysteine residue in the GluN1 subunit.

### Cross-linking partners in the L1-L1 interface

**Figure 3:**
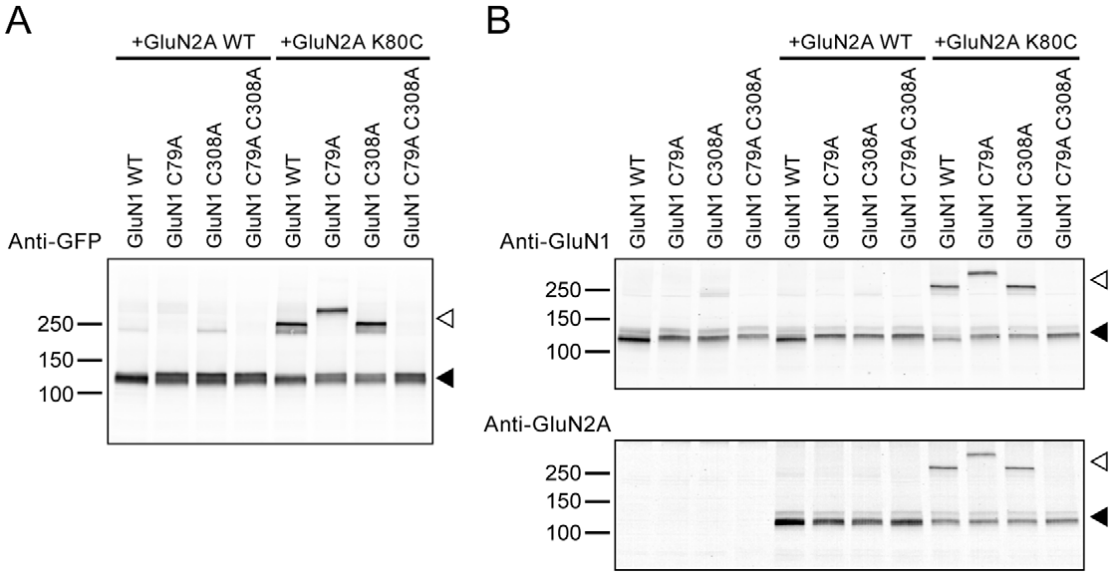
Cross-linking partners on the GluN1 ATD at the L1-L1 interface. Western blotting analysis of GluN1 cysteine knockout mutants coexpressed with GluN2A WT or GluN2A cysteine mutants. (**A**) The blot probed by GFP antibody. (**B**) The blot probed by GluN1 and GluN2A antibody. Open arrowhead indicates the cross-linked dimer bands. Filled arrowhead indicates positions of GluN1 or GluN2A monomer bands.

We next sought to identify the cross-linking partner of GluN2A K80C. In the GluN1 ATD there are three endogenous cysteines: C22, C79 and C308. Among them, C79 and C308 are within or near the L1 interface (Fig. S1) and are predicted to form an intra-subunit disulfide bridge based on the GluA2, GluK2 and GluN2B ATD crystal structures [11], [12], [13], [15]. Nevertheless, we mutated these residues to alanine and coexpressed them with the GluN2A K80C subunit. Neither the C79A nor the C308A single mutant was sufficient to prevent the dimer formation. However, when both cysteines were eliminated, GluN2A K80C no longer cross-linked with GluN1 (Fig. 3A). This suggested that both GluN1 C79 and C308 could serve as a cross-linking partner of GluN2A K80C. The shift of the dimer band in the GluN1(C79A)/GluN2A(K80C) combination may reflect a different ATD conformation adopted by the dimer cross-linked between GluN1 C308 and GluN2A K80C, leading to a change in mobility. The identification of partner residues on GluN1 ATD confirmed that GluN2A ATD associates with GluN1 ATD. We also showed that both GluN1 and GluN2A were present in the cross-linked dimer by probing with GluN1 and GluN2A antibody (Fig. 3B). Taken together our data is consistent with the conclusion that the NMDA receptor ATD forms a heterodimer.

### GluN1 and GluN2B ATD heterodimer

**Figure 4:**
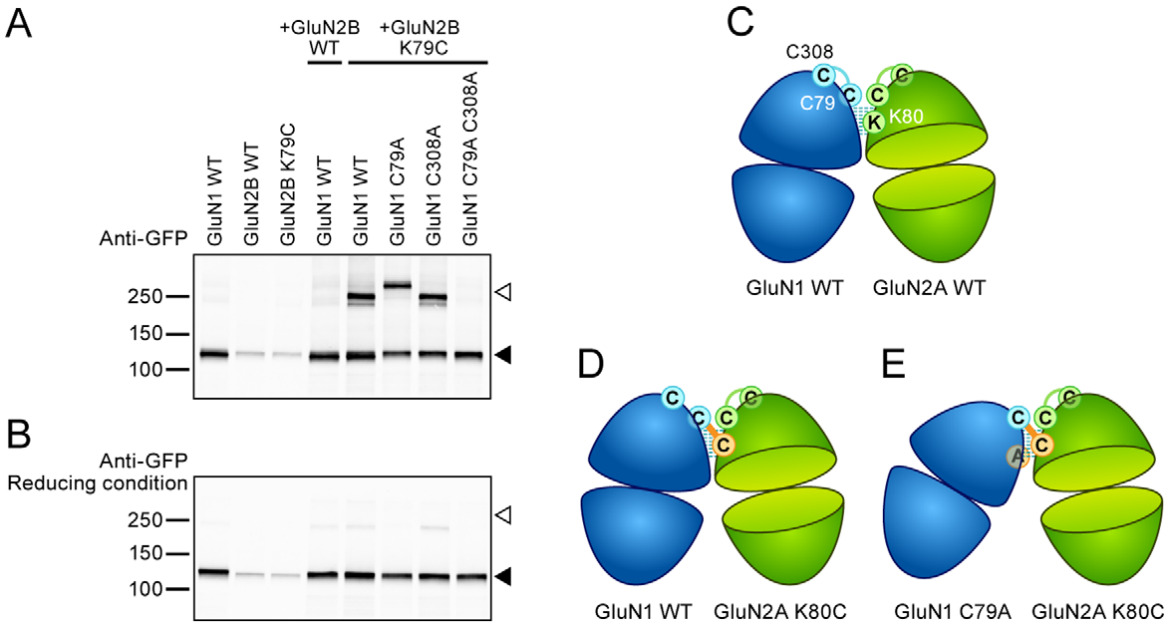
GluN2B ATD forms a heterodimer with GluN1 ATD. Western blotting analysis of GluN1 cysteine knockout mutants coexpressed with GluN2B WT or GluN2B cysteine mutants probed by a GFP antibody. (**A**) Non-reducing conditions. (**B**) Reducing conditions. Open arrowhead indicates the cross-linked dimer bands. Filled arrowhead indicates positions of GluN1 or GluN2B monomer bands. The predicted molecular weights of GluN1 and GluN2B construct are both ∼125 kDa. (**C**) Cartoon model of NMDAR ATD heterodimer. In wild-type subunits, the endogenous cysteines form intra-subunit disulfide bonds. Interactions between the L1-L1 interface are emphasized by thin lines. (**D** and **E**) A summary of cross-linking results in the current study. GluN1 C79A and GluN2A K80C residues are colored in orange. The disulfide bond between endogenous cysteines of GluN1 and engineered cysteines of GluN2 is represented by thick orange lines. For clarity, in (**C** and **E**) only GluN2A is drawn, but our results suggest that GluN2B shares a similar orientation in terms of ATD heterodimer. The equivalent residue of GluN2A K80 in GluN2B is K79.

Native NMDA receptors are assemblies of the GluN1 subunit with various GluN2 subunits and the GluN2 ATD is responsible for determining the distinct receptor properties in different subtypes of NMDA receptor [17], [18]. Thus, we asked whether a subunit different from the GluN2A could form a similar disulfide bond-mediated ATD heterodimer with the GluN1 subunit. If it did, then it would suggest conservation in the L1-L1 heterodimer interface among different subunit combinations. To test this hypothesis, we made the GluN2B K79C mutant and coexpressed it with wild type GluN1. Analyses by Western blotting showed the formation of a putative GluN1–GluN2B dimer band, similar in mobility to that formed by the GluN1/GluN2A subunit combination (Fig. 4A). The putative dimer band was redox-sensitive, disappearing in the presence of DTT (Fig. 4B) and consistent with the idea that subunits were linked by disulfide bonds. This finding supported the notion that both GluN2A and GluN2B subunits can form a local ATD heterodimer with the GluN1 subunit through contacts in the L1-L1 interface.

## Discussion

The introduction of cysteine residues is a widely accepted method to probe neighboring molecular contacts of a protein [20]. Two cysteine residues will form a cross-link only when they are in close proximity [20], [21]. We identified a cysteine mutant of GluN2 ATD that was able to spontaneously cross-link with endogenous cysteines C79 and C308 on GluN1 ATD, uncovering the interaction between heteromeric ATD subunits (Fig. 4C). When we mutated residues C79 and C308 to alanine, specific cysteine residues in GluN2 ATD no longer resulted in inter-subunit cross-links. Importantly, experiments by other groups have shown that GluN1 C79A and C308A mutants coexpressed with GluN2A traffic to the cell membranes, form functional receptors [5], [22] and do not affect receptor oligomerization [9]. Thus, the reason why we did not observe the formation of disulfide bond-mediated dimers with GluN1 C79A C308A double mutant (Fig. 3 and 4) is not due to the impairment of subunit expression, trafficking or assembly. By contrast, our data strongly suggest that the GluN1 residues C79 and C308 directly participate in forming inter-subunit disulfide cross-links. The fact that GluN2A K80C or GluN2B K79C could cross-link with more than one GluN1 residue (Fig. 3 and 4) suggests that the L1-L1 interface is somewhat dynamic or plastic. This observation opens up the possibility that rearrangements within the L1-L1 interface occur during gating of the NMDA receptor. Compared to GluN1(WT)/GluN2A(K80C), the band shift in the GluN1(C79A)/GluN2A(K80C) complex but not in the GluN1(C308A)/GluN2A(K80C) combination (Fig. 3 and 4), implies that although both GluN1 C79 and C308 could serve as cross-linking partners, GluN2A K80C preferentially cross-links with GluN1 C79 when coexpressed with wild type GluN1 (Fig. 4D). Based on available structural information [15], the NMDA receptor ATD also adopts a “clamshell” conformation. It has been proposed that NMDA receptor ATD clamshells spontaneously switch back and forth between open/closed-cleft conformation and tune the properties of NMDA receptors [17]. The rearrangement of L1-L1 interface may participate in this process and make it possible for the remaining GluN1 cysteine to form disulfide bonds with the engineered cysteine on GluN2 when one of the GluN1 cysteines has been knocked out (Fig. 4E).

Although we did not exclude the possibility that the L2-L2 interface is also responsible for dimerization, our results suggest a different role for the L2-L2 interface in the NMDA receptor ATD compared to GluA2 or GluK2 ATD. In non-NMDA receptor ATD, the dimer is held together by extensive contacts between the L2-L2 and L1-L1 interfaces [11], [12], [13]. Indeed, the GluK2 ATD L2-L2 interface mutant, L151C, forms spontaneous cross-linked dimers [19], but similar mutants in GluN1 and GluN2A ATD did not (Fig. 1B and C). The lack of this interaction in the NMDA receptor may be because NMDA receptor ATD adopts a twisted conformation, as shown by the structure of isolated GluN2B ATD [15] and by the cross-linking experiments on GluN2A ATD [23]. At present, there are no specific ligands for the ATDs of non-NMDA receptors, and the stabilization of L2-L2 interface in GluK2 ATD by cross-linking has minimal effects on channel properties [19]. By contrast, the GluN2 ATD is the target for the physiological ligand Zn^2+^ or for pharmacological compounds, which allosterically regulate the function of the receptor [1], [2]. We speculate that the difference in the L2-L2 interface between NMDA and non-NMDA receptor helps explain how ATD ligands modulate the functional properties of NMDA receptors.

The GluN1 ATD has been proposed to mask the retention signal on the GluN2A ATD [24], a conclusion that is in line with the heteromeric interaction of their respective ATDs. Our results may also explain why the isolated GluN2B ATD predominately exists as a monomer when the GluN1 ATD was not coexpressed [15], [25], [26]. In fact, the isolated GluN2 ATD could associate with the isolated GluN1 ATD in solution [25], [26]. On the other hand, several studies have shown that full-length GluN1 subunits or isolated GluN1 domains, when expressed alone, could form homodimers [6], [9], [25], [26], [27], [28], [29], [30]. This has been used as an argument supporting the dimer-of-homodimer model for NMDA receptor. In the present study, upon expression of the GluN1 subunit alone, we also detected a weak band with a size approximately commensurate with a GluN1 homodimer (Fig. 1A). We suggest the appearance of this band likely represents the extent to which GluN1 subunits have a weak propensity to form homodimers. Even though this observation is consistent with homodimerization of the GluN1 subunit, it does not mean that the GluN1 subunits form a homodimer in an intact NMDA receptor. Here we studied the association of ATDs within intact NMDA receptors and we found that ATDs form local heterodimers.

The heteromeric NMDA receptor ATD is reminiscent of the GluA2 ATD in the GluA2 full-length structure [14]. The full-length structure revealed two “non-equivalent” pairs of subunits, A/C and B/D where the two GluA2 ATD dimers are composed of two conformationally distinct subunits A–B and C–D. Based on the GluA2 full-length structure, a model of NMDA receptor architecture has been proposed which suggested a dimer-of-heterodimer assembly with a GluN1–GluN2-GluN1–GluN2 orientation [14]. Our results are in harmony with this model in regard to the heterodimeric feature of NMDA receptor; however, our results were not restricted to the GluN1–GluN2-GluN1–GluN2 orientation proposed by the model. In the NMDA receptor, ATD heterodimers could also be arranged in a GluN1-GluN1-GluN2-GluN2 manner, although in this case the two ATD heterodimers will be arranged ‘in parallel’, an arrangement that precludes an overall two-fold axis of symmetry. Further experiments, such as the cross-linking of residues in the inter-heterodimer interface [14], [19], are needed to clarify this issue.

## Materials and Methods

### Receptor expression

We used the rat GluN1a splice variant construct (accession number NP_058706, hereafter referred to as GluN1) with a deletion from M848 to S938 [31], the rat GluN2A construct (NP_036705) with a deletion from I867 to V1464, and the rat GluN2B construct (NP_036706) with a deletion from I868 to V1482 [32]. These carboxyl terminal truncations reduce non-specific cross-linking yet when coexpressed they assemble to a receptor with wild type function [33], [34], [35], [36], [37]. All constructs have green fluorescent protein (GFP)-tags at their carboxyl termini. The constructs were expressed in tsA201 cells (HEK 293T, ATCC CRL-11268) plated in 40 mm dishes at a density of 5×10^5^ cells/ml the night before the transfection. Cells were transfected with lipofectamine 2000 (Invitrogen) according to the manufacturer's instruction. For expression of a single subunit, 1 µg of DNA per dish was used for transfection; for the co-expression of GluN1 and GluN2 subunits we employed a total of 2 µg of DNA with a GluN1∶GluN2 plasmid ratio of 1∶1. The DNA concentration was estimated by absorbance at 260 nm (A_260_). At 5 hours post-transfection, the Opti-MEM medium (Invitrogen) was replaced with fresh medium containing 200 µM of dichlorokynurenic acid and 2-amino-5-phosphonopentanoic acid. Cells were harvested at 24 hours post-transfection. The efficiency of transfection was estimated by GFP-dependent epifluorescence.

### Site-directed mutagenesis

 The carboxyl terminal-deleted ‘wild type’ rat GluN1, GluN2A and GluN2B subunits were used as templates and mutations were introduced into desired positions by QuikChange II site-directed mutagenesis kit (Agilent) and confirmed by DNA sequencing of both strands of the entire NMDA receptor open reading frames. Here, the numbering of amino acid residues starts at the position of the initiation methionine. When referring to other studies, however, the numbering follows the original, cited literatures and, in some cases, the numbering may start at the first residue of the mature protein.

### Western blotting

Transfected tsA201 cells were solubilized in 200 µl of lysis buffer (150 mM NaCl, 20 mM Tris, 40 mM *n*-dodecyl-β-D-maltoside, 1 mM phenylmethylsulfonyl fluoride, and a protease inhibitor cocktail composed of 2 mg/ml leupeptin, 0.8 mM aprotinin, and 2 mM pepstatin A, pH = 8.0) for 1 hr. These whole-cell extracts were centrifuged for 40 minutes at 40000 rpm at 4°C and the supernatants were collected. The proteins in the collected supernatants were separated by sodium dodecyl sulfate polyacrylamide gel electrophoresis and approximately 4–6 µg of total protein from whole-cell extracts were loaded onto 4–15% gradient gel per lane. The protein concentration was estimated by absorbance at 280 nm (A_280_) and corrected by the equation: protein concentration (µg/µl) = (1.55×A_280_)−(0.76×A_260_) [38]. For reducing conditions, a final concentration of 100 mM dithiothreitol (DTT) was added to samples before they were loaded onto the gels. Proteins were then transferred to a nitrocellulose membrane and probed with primary antibodies. The anti-GFP (Invitrogen, A11122), anti-GluN1 (Millipore, MAB1586) and anti-GluN2A antibody (Invitrogen, 480031) were used at dilutions of 1∶5000, 1∶500 and 1∶1000, respectively. The secondary antibodies (LI-COR IRDye 680, 926-32220 and IRDye 800CW, 926-32211) were used at a dilution of 1∶10000.

## Supporting Information

Figure S1 **ATD residues studied in this work.** Here we mapped GluN1 and GluN2A residues onto the GluN2B ATD structure [15] based on a multiple amino acid sequence alignment. This figure is for the demonstrative purpose to show the possible location of these residues and it does not represent an accurate structural model. Endogenous cysteines of GluN1 are dark blue and the residues studied in this work are orange.
(TIF)
