# Supplementary figures and images for: Amino Terminal Domains of the NMDA Receptor Are Organized as Local Heterodimers
Source: PLoS One. 2011 Apr 22;6(4):e19180. doi: 10.1371/journal.pone.0019180 (PMC3081335; doi:10.1371/journal.pone.0019180)

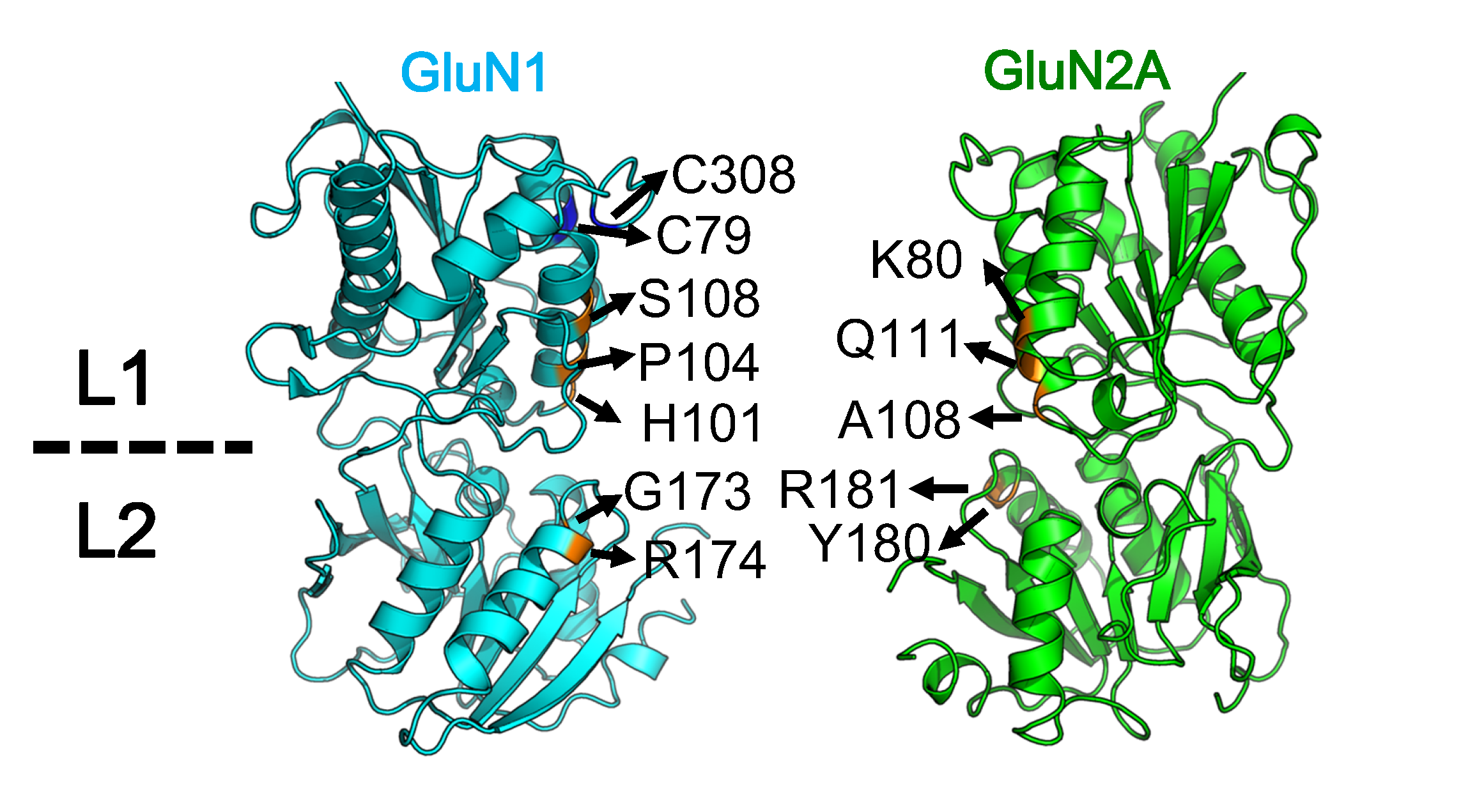

Supplement: Figure S1 — ATD residues studied in this work. Here we mapped GluN1 and GluN2A residues onto the GluN2B ATD structure [15] based on a multiple amino acid sequence alignment. This figure is for the demonstrative purpose to show the possible location of these residues and it does not represent an accurate structural model. Endogenous cysteines of GluN1 are dark blue and the residues studied in this work are orange. (TIF) [file pone.0019180.s001.tif]
